# Health visitor and community health nurse perspectives of supporting parents caring for unsettled babies: a qualitative interview study

**DOI:** 10.1136/bmjopen-2025-101051

**Published:** 2026-02-12

**Authors:** Lucy Smith, Samantha J Hornsey, Sue Latter, Amy Dobson, Sascha Miller, Kate Henaghan-Sykes, Sue Adams, Miriam Santer, Ingrid Muller

**Affiliations:** 1 Primary Care Research Centre, Faculty of Medicine, University of Southampton, Southampton, UK; 2 School of Psychology, Faculty of Environmental and Life Sciences, University of Southampton, Southampton, UK; 3 Human Development and Health, Faculty of Medicine, University of Southampton, Southampton, UK; 4 School of Health Sciences, University of Southampton, Southampton, UK; 5 Solent NHS Trust, Southampton, UK

**Keywords:** Clinical Decision-Making, EDUCATION & TRAINING (see Medical Education & Training), Health Services, Health Workforce, Nurses, Community child health

## Abstract

**Abstract:**

**Objectives:**

The aims of this study were to explore how health visitors (HVs) and community health nurses (CHNs) assess unsettled baby behaviours, how their perceptions of these behaviours influence decisions about support offered, and how able they feel to deliver support to families of unsettled babies.

**Design:**

Qualitative semi-structured interviews were conducted, recorded and transcribed. Data were analysed using Reflexive Thematic Analysis.

**Setting:**

Potential participants were invited nationally via social media and via Health Visiting Service managers from an NHS Trust. Interviews took place remotely.

**Participants:**

17 HVs and 3 CHNs were purposively selected to include a wide range of perspectives.

**Results:**

Three themes were developed, (1) HVs’ perceptions of parents’ sense-making which explains how HVs/CHNs understand parents’ beliefs around unsettled babies; (2) care pathway which highlights the importance HVs place on creating emotional space for the baby, the parent and the health visitor within the pathway (containment); and (3) service delivery decline, which outlines the impact of funding cuts to the services on the HVs’ ability to provide support for families. Lastly, a new concept – the Tipping Point model - was created to holistically conceptualise the experiences of HVs providing support for unsettled babies in the UK.

**Conclusions:**

Policy makers need to organise services to value and support the role of the health visiting team in ‘containment’. HVs identified a training need around assessing and advising about unsettled babies to place them in a stronger position to support families. Further research is needed into different models of support for families of unsettled babies from the wider primary care team and/or from digital services.

**STRENGTHS AND LIMITATIONS OF THIS STUDY**

- In-depth interviews gathered rich data from a diverse range of healthcare practitioners working across a range of English regions.
- A more ethnically diverse sample and the inclusion of more community health nurses would have enhanced the applicability of the findings regarding views of health visiting teams.
- Social media recruitment may have reduced participant diversity or attracted those with particular interest in the topic.

## Introduction

 Parents and caregivers (hereafter referred to as parents, to include carers) of babies in the first year of life commonly report excessive crying^1^ or other unsettled baby behaviours or symptoms^2^ such as vomiting, rash, constipation and diarrhoea. These can have negative impacts such as increased stress and anxiety; bonding difficulties^3^; risk of non-accidental injury,^4^ unwanted changes to breastfeeding/chest-feeding (hereafter referred to as breastfeeding, to include chest-feeding)^5^; and inappropriate medication.^6^ A recent systematic review of 22 quantitative studies suggested baby crying and ‘fussing’ are linked to changes in feeding decisions such as reduced breastfeeding confidence, motivation, duration and introduction of commercial formula.^7^ Within this paper, excessive crying is defined as crying that continues for longer than usual for the baby, after repeated attempts have been made by the caregiver to meet their needs. Excessive crying or other infant symptoms are often interpreted as cow’s milk allergy (CMA) or gastro-oesophageal reflux; however, there is growing concern that this may cause significant harm to babies and families; overtreatment of symptoms can undermine breastfeeding, lead to added stress and anxiety for the family, and increase risk of negative outcomes and discomfort for the child.^8 9^

Large scale cohort data suggest that CMA is often inaccurately diagnosed,^10^ with a prevalence of less than 1% in babies, but up to 40% being labelled with CMA.^11 12^ Reflux is common, with estimates that symptoms occur in up to 50% of babies under 2 months old, and up to 67% in 4-month-olds,^13^ but many can be self-managed with high quality feeding support.^14 15^ A label of CMA and/or reflux has been linked to significant resource use and costs to the National Health Service (NHS)^16^,^18^ with many parents receiving a prescription for medication,^19^ with associated side effects.^20^ Overmedicalisation or mislabelling of symptoms as CMA or reflux can have implications for the parent and baby. For example, perceptions of CMA for breastfeeding parents can result in dietary exclusions, unwanted reduction of exclusive breastfeeding to combination feeding, early cessation of breastfeeding entirely and introduction of specialist formula milk unnecessarily.^15 21^ Current guidelines advocate for dietary elimination; however, evidence for the transfer of allergens through breastmilk, enough to trigger a reaction, is limited and insufficient to support dietary changes.^15 22^ Unwanted changes to breastfeeding can also be associated with decreased self-confidence and poorer mental health, including potential for significant grief and trauma.^15 23^ New guidelines recommend more conservative management options.^5^

A recent systematic review^24^ of parental perceptions of excessive infant crying identified an evidence gap regarding how parents assess symptoms, where they seek support and the process by which they come to label symptoms as CMA or reflux. Recent qualitative research aimed to address this gap by exploring parental perceptions of common baby symptoms such as excessive crying, how parents assess symptoms, seek help and the impact of healthcare professional (HCP) support on families’ perceptions of baby symptoms, feeding decisions and coping.^25 26^ However, little is known about HCP perceptions and experiences of assessment and supporting families with unsettled babies.

In the UK, health visiting teams are the universal contacts for parents with children aged 10 days to 5 years.^27^ The role is important in supporting families with unsettled baby behaviours.^28^ However, health visiting teams are currently significantly under-resourced and understaffed, and supporting the workforce is a national priority.^29^

### AIMS

This study aimed to explore how health visiting teams assess unsettled baby behaviours such as crying and vomiting, how their perceptions of baby behaviours influence their decisions about the care they offer, and how well health visitors (HVs) feel they are supported to deliver this care.

## Methods/methodology

This study is reported using the Consolidated Criteria for Reporting Qualitative Research.^30^

### Design

Qualitative semi-structured interviews were conducted with HVs and community health nurses (CHNs) about experiences of supporting families with unsettled babies.

### Study setting and recruitment

The study was advertised to potential participants via private social media groups for HVs and via HV team managers in a local NHS Trust. Study adverts had researcher contact details and linked to online study information and expression of interest form so that HV/CHNs could inform the research team directly that they would like to take part. In the recruitment material, we gave some brief examples of behaviours/symptoms unsettled babies may have (eg, ‘excessive crying’, ‘vomiting’, ‘fussing unless being held’). This helped us to recruit eligible health professionals; however, due to a lack of clear conceptual definition of this concept, we did not give a specific definition of what constitutes an unsettled baby. Each participant received a £25 voucher for their contribution to the study. We are not aware of who decided not to express an interest because we did not know who the study was advertised to unless they expressed an interest to us after seeing the recruitment materials.

### Sample

Participants were purposively sampled to ensure a range of age, years in practice, region and demographics of families they support. It was anticipated that 20–25 interviews would provide sufficient information power^31^ for interpretation of the data. After 20 interviews were conducted, this point was considered to have been reached, with no new major themes being developed.

### Inclusion and/or exclusion criteria

Any HV or health visiting team member who had supported families with an unsettled baby in the past 12 months was eligible. Other HCPs, service managers and non-practising HVs were excluded.

### Data collection

Interviews took place between October 2023 and March 2024. LS (a female postdoctoral researcher with experience in qualitative research methods) conducted the interviews and did not know the participants prior to the interview and presented herself as neutral and non-clinical to the participants. Interviews were carried out remotely to maximise regional diversity within the sample. Remote interviews also offered flexibility to take part in the study around the working day. One interview took place via telephone or videocall. There were no non-participants present at the interview.

A semi-structured topic guide (online supplemental file 1) was developed collaboratively by the research team (comprising clinical, professional, academic and public contributor expertise). Six main questions asked about (1) beliefs about what the term unsettled baby means to them, including beliefs around unsettled babies’ behaviour in relation to symptoms, causes, assessment and treatment; (2) experiences of supporting families with unsettled babies; (3) beliefs about the role general practitioners (GPs) play in supporting families with unsettled babies; (4) experiences of working collaboratively with GPs; (5) levels of confidence and/or training the HVs/CHNs received to enable them to support families with unsettled babies and (6) beliefs around any unmet needs for families with unsettled babies. All interviews were audio-recorded, professionally transcribed verbatim and anonymised. Notes were recorded by the interviewer during the interview which were further refined in an interview summary sheet after each interview took place.

### Data analysis

All interview transcripts were analysed inductively using Reflexive Thematic Analysis.^32^ This allowed for a flexible and pragmatic approach to data analysis with the paradigmatic framework of constructivism and interpretivism. Data were handled using NVivo V.14 software. LS carried out the initial coding framework and all coding was discussed with all members of the team, who fed back. LS iterated the coding based on feedback, and this was again circulated to the whole team for input. The team read the interview summaries, and IM (female, professor and qualitative expert), AD (female, PhD student and HV, qualitative researcher) and SJH (female, postdoctoral researcher, experienced qualitative researcher) each further read two separate transcripts to aid development of coding, interpretation and analysis. The team was happy with the codes/themes, developed through team discussion and in partnership with public contributors. It was not within the capacity of this project to return transcripts/themes to participants for checking.

### Ethical considerations

This study was reviewed and approved by the University of Southampton Research Governance office (ERGO reference 82667) and the Health Research Authority IRAS reference 329794. In advance of the interview, participants were emailed study information, given the opportunity to ask questions and sent a consent form to consider prior to taking part. Verbal consent was then recorded at the start of each interview.

### Patient and public involvement

Public contributor and co-author KHS was involved in all aspects of the research, including study design, securing funding, attending research team meetings and data analysis and interpretation. Three HVs (two practising and one retired) helped to shape the interview topic guide.

LS, IM and KHS discussed key study findings with a parent group from a local children’s centre, where all eight parents had lived experience of unsettled babies. Feedback from this group was that parents felt isolated and would value more accessible services, especially access to HVs which they found increasingly challenging due to a perceived shortage of HVs. The input from this group helped support our analysis and reinforced key findings around service delivery, as parents’ perspectives closely reflected the professionals’ experiences.

### Findings

31 HV team members expressed interest in participating. Purposive sampling was used to ensure a diverse sample in terms of professional role, age, years in practice, region and demographics of families supported. Table 1 shows the sample demographics. Interviews were conducted with 20 HVs and CHNs, with average interview duration 50 min (range 40–75 min).

**Table 1:** Sample characteristics

		N (%)	Mean (range)
Gender	Female	20 (100)	
Age in years			43.8 (29–66)
Ethnicity	White British	100	
Years in practice			9.35 (0–40)
Professional role	HV	17 (85)	
	CHN	3 (15)	

CHN, community health nurse; HV, health visitor.

**Figure 1:**
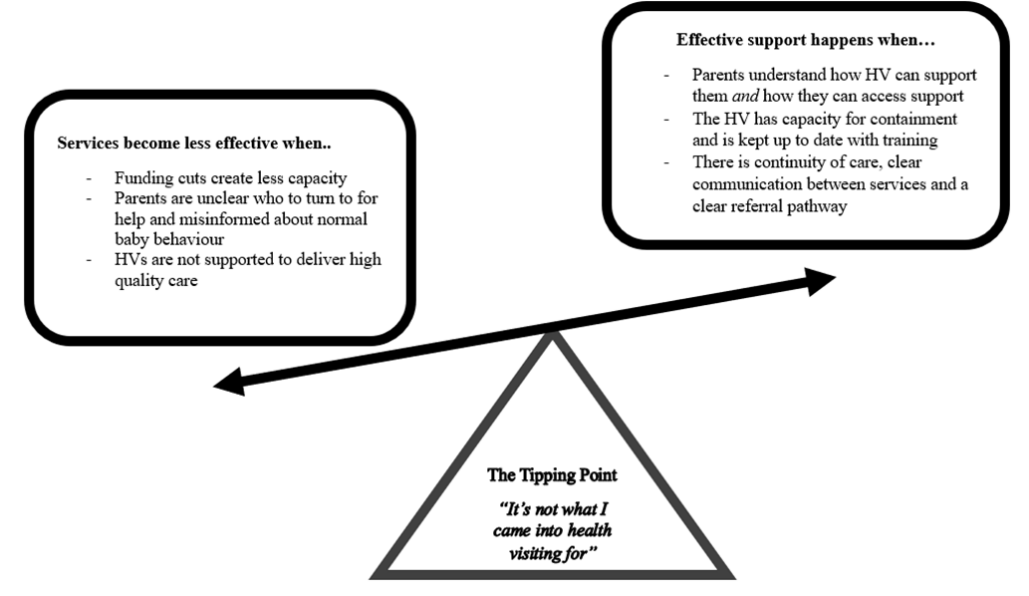
The tipping point model. HV, health visitor.

Three themes were developed, described below. These themes were then contextualised within an overall ‘tipping point model’ (figure 1), which was developed alongside our data analysis to illustrate how these themes interact to create an HV service which is currently struggling to provide effective support for families. Table 2 shows the breakdown of themes and subheadings.

**Table 2:** Themes and subthemes

Theme	Subthemes
HVs’ perception of parents’ sense making	Pressure for a ‘quick fix’ Sociocultural context and commercial influences Families’ feeling of failure Families’ difficulties in navigating services
Challenges to care pathways for unsettled babies	Assessing unsettled babies is complex Needing time to practise ‘containment’ Signposting Problems caused by service constraints
Stretched services with changing priorities	Impact of funding cuts on service quality Support within the health visiting team Interprofessional relationships

HVs, health visitors.

### HVs’ perception of parents’ sense-making

The HV and CHN interviewees described their experiences of talking to parents about how they made sense of their baby being unsettled, considering causation, treatment and the wider contextual factors which may shape this.

#### Pressures for a ‘quick fix’

Many of the health professionals interviewed perceived that parents often had unrealistic expectations about what normal baby behaviour was, with some wanting a ‘quick fix’ to stop their baby being unsettled. While HV staff understood the strain parents were under with dealing with such symptoms, they also reported that this led to the parents either phoning the HV team duty desk or contacting the GP seeking a solution. Participants felt that in doing this, parents were misinterpreting normal baby behaviour (referred to by several HVs as the ‘fourth trimester’) as problematic.

I think sometimes parents, rightly and understandably, want a quick fix, and they want there to be a definitive answer.

(P10, HV)

#### Sociocultural context and commercial influences

Contextual factors seen as influencing parental sense-making were technology (use of the internet), familial pressure, cultural expectations about normal baby behaviour and societal norms about a ‘good baby’, which may have contributed to the need for a ‘quick fix’. Some participants believed that formula companies took advantage of exhausted and vulnerable families. Technology was viewed as a potentially useful platform through the use of apps or approved websites, but also as fuelling a societal norm that babies are settled, and parents should be able to easily ‘cope’. Participants also spoke about having to compete with misinformation provided on social media platforms:

But if we don’t provide that, that’s where they’re going to go to TikTok and all these other things that actually give them an unrealistic expectation. Celebrities and all of this sort of stuff. It’s just not helpful for most people.

(P4, HV)

The influence of peers with their own children was often viewed as a potential source of misinformation for parents too:

Definitely sometimes it’s someone might have said to their friend, ’Oh, they’re really unsettled,'and the friend will just, quite well-meaningly, say, ‘Oh, maybe they've got an allergy.’ (P12, CHN)

Some participants discussed culture and felt that families from non-Western cultural backgrounds did not present as often with unsettled babies. When asked, participants felt there were different cultural norms in terms of help-seeking, expectations around baby crying and parenting practices. Having extended familial support was seen as key.

Ethnicity is a big one. In central [City] there’s a lot more ethnicities, in central [City], and they have got… Not all the time, but some families I’ve gone to there’s generations living there. It’s like the father’s mother and their mother.

(P11, HV)

#### Families’ feeling of failure

Many of the health professionals interviewed had personal experience of an unsettled baby and real empathy for parents, who were perceived by the HVs as often feeling like a failure for not being able to soothe their baby.

Obviously, because I was that breath of fresh air, and I didn’t have all the hormones and hadn’t been up all night with him, he very usually would settle as soon as he was handed to me, which is awful for those parents because they then—it reinforces this negative self-belief that they are the issue, so she would say, 'He’s okay, he’s okay for you,'and I was very, really try and explain it.

(P8, HV)

#### Families’ difficulties navigating services

Participants expressed concern that many families were being passed between services with no clear guidance on who to contact. The lack of drop-in clinics (which participants report have not been reintroduced post-COVID-19) means parents have to take responsibility for scheduling appointments with the GP or the HV, and this may marginalise those families who are less likely to engage with services.

The people that turn up at A&E sometimes are those very anxious parents that maybe haven’t got that support, and they just want to know something about reflux or weaning. Actually, they’re turning up for a really acute service, when actually they could have benefited quite easily from just turning up at a clinic if they wanted to.

(P14, CHN)

### Challenges to care pathways for unsettled babies

Participating HVs and CHNs discussed experiences of assessing, supporting, signposting and/or referring families with unsettled babies or suspected CMA or reflux for further investigation.

#### Assessing unsettled babies is complex

Participants generally described assessment as a ‘challenging’ process, with careful judgement accompanied by watching and waiting to see how the baby developed, especially over the first 12 weeks when most problems were reported. There was often no single answer for why the baby was unsettled, and lack of clarity about identifying problems, which left participants uncertain.

It’s a tricky one because we get this in health visiting a lot, about a baby crying, and it’s really hard to unpick because babies do cry, and it is normal for a baby to cry. I suppose it’s trying to work out whether that’s normal or not normal.

(P11, HV)

#### Needing time to practise ‘containment’

Participants described how they treat an unsettled baby by listening to the parent to understand their perspective. Participants felt that in the majority of cases, the unsettled behaviour was actually normal baby behaviour, and parents needed reassurance, empathy and a space to express their concerns and feel listened to; this is referred to in the literature as ‘containment’.^33^ HVs spoke of ‘listening visits’ and constructed a key part of their expertise as being a vessel to hold the emotions of the parents in the moment, so they could express and process how they were feeling, showing containment:

it’s about trying to reassure and listen, but whilst trying not to I guess give false promises of things will be okay, and by next week things should have settled, and just let them know that you’re listening.

(P10, HV)

For breastfeeding families, where CMA was not suspected, participants felt that their role was partly about providing reassurance. Parents were reassured that their baby was getting enough milk to build confidence in feeding and were often referred to breastfeeding clinics.

We put the pathway in place to really look at how we could protect any breastfeeding. We have some mums who wanted to re-lactate following education.

(P17, HV)

Containment was seen by the majority of participants as an important part of their role, but difficult to deliver in the context of current pressures on the HV service. Participants described how cutbacks and service reorganisation restrained continuity of care due to time restrictions and staff capacity, undermining the relationships key to containment:

So our discretion as to how often we can visit and what support we can put in is now very much stricter, and quite a corporate KPI-led way of managing the team, and I feel that’s based on staffing levels, really.

(P13, HV)

Services were described by participants as being ‘reactive’ in recent years as opposed to holistically supporting families, therefore, HVs felt that their expertise in containment for the parent may not be recognised leading to under-utilisation of HV support:

I do sometimes think that the health visiting job, our role has been forgotten. I don’t know. When you go into homes and things, they’re like, ‘Oh, I didn’t know what a health visitor did.’ (P2, HV)

#### Signposting

Participants discussed how they provided information that they thought would be useful for parents via website links or leaflets. Participants would refer to other services to support the family, such as the infant feeding specialist service or breastfeeding clinics, expressing frustration that all they could really do was signpost.

“I don’t think they think very highly of us because a lot of the time, they’ll ring up and we’ll reassure or we’ll signpost as well. We can do referrals, but often we are that person in the middle, that signposting to different services.”

(P11, HV)

#### Problems caused by service constraints

Some HVs said they thought that GPs overprescribe medications or specialist formula milk due to time constraints in the appointment and service pressure. Most participants felt that CMA/reflux was overdiagnosed, as parents often went straight to the GP, often at a time of crisis, bypassing the HV and therefore the opportunity for containment.

It can be difficult, and a lot of the time they will just prescribe [medication] or lactulose, either a laxative or an anti-reflux, just that quick initial… They’ve only got 15 minutes for their appointments.

(P11, HV)

Some participants felt that GP service constraints created more work for the HV, as lack of follow-up or continuity of care meant the baby may experience side effects of medicines prescribed or become more unsettled, meaning the HV had to work with the family again to manage these developments.

They [GP] often won’t invite them back in for a review appointment because of their capacity. They physically haven't got the capacity or the staffing, whereas maybe we’ve got more opportunity to go back.

(P11, HV)

### Stretched services with changing priorities

#### Impact of funding cuts on service quality

HVs/CHNs constructed their experience of working within a service which is often changing, underfunded and in recovery following the COVID-19 pandemic. Funding cuts were viewed as underpinning many of the issues with inter-professional relations and the capacity of the HV team to provide containment for the family. Almost all drop-in clinics were stopped during COVID-19, and participants reported that most have not been reinstated. Participants felt under pressure to meet targets and/or to justify all their patient contacts, sometimes with their line manager.

Often you’re agenda-matching as well. Some parents, they want to talk about, quite rightly, what they want to talk about, so you’re trying to agenda-match that.

(P11, HV)

HVs described how the nature of their role is now completely different—primarily caring for families with complex social needs, with other team member nurses providing the universal care that HVs were originally associated with.

My understanding of the role of the health visitor, I guess, has changed quite dramatically in terms of the capacity that health visitors have now, in terms of doing things and obviously the workload that’s allocated to a health visitor versus the workload that’s allocated to our band five nurses within the team.

(P10, HV)

Consequently, several participants described that HVs are leaving the NHS in large numbers, adding more pressure on the capacity of staff who remain in post, with many reportedly burning out. Some HVs described no longer being able to use expertise within the team, through the informal learning that can occur when colocated.

Rather than recruiting for another health visitor, they’re kind of like spreading that caseload and putting more on to the ones that are staying. I think the sickness rate, because of that and because of the responsibilities increasing, has gone up. I think you've seen a lot of people wanting to reduce their hours.

(P12, CHN)

When you’re in an office and you’re doing a phone call, you get people say, 'Oh, that was interesting. I didn't think about that,’or, ‘Have you considered this, because I didn't hear you mention that?’That’s really good for growth as a professional and changing your practice, but there’s less of that now with all the virtual working.

(P16, HV)

Some HVs also reported feeling lonely due to the isolation of lone working, and there was a sense that camaraderie was being lost:

We had a WhatsApp group with the six of us and if we couldn’t fit something in, one of the other people would pick it up, ‘Actually, I’m really struggling, can someone do this for me?’and you’d always do it, and I don’t have that any more. It’s a lot, and I’m covering the whole [area]. I haven’t got a locality now, which I did before.

(P12, CHN)

The construction of being an HV within the context of a service facing funding cuts and staff shortages appeared to leave HVs feeling conflicted in their role. Many were conscious of the fact that they were not able to give families the support they knew they could provide to enhance their lives with an unsettled baby, leading to feelings of frustration:

It’s not what I came into health visiting for but I’m hoping when the financial situation’s better, we take on more staff, we can go back to that way because families need that.

(P15, HV)

#### Support within the health visiting team

All participants reported that they felt well cared for within their team, and there was a feeling of a ‘shared struggle’ which created a sense of team membership. Supervision meetings were very vital to discuss cases, or as a space for HV containment.

The team are great. So, they have a team group, and you can just check in and pop a question into the chat and somebody will come back to you, hopefully…

(P10, HV)

Aside from supervision, many felt it would be useful to have a module in their HV qualification training which was focused on infant feeding problems and crying. Most HVs reported learning on the job, and through gaining experience.

It would be very helpful to have some sort of course on, specifically, unsettled babies, because a lot of it comes into other aspects of care… there’s nothing really specific.

(P14, CHN)

#### Interprofessional relationships

HVs/CHNs discussed their relationships with other health professionals as being less collaborative than they would like and less collaborative than prior to service restructuring. They spoke about wanting a more integrated systems approach to supporting parents/carers with unsettled babies. HVs used to be localised to a GP surgery, facilitating effective interdisciplinary communication. Now HV teams spoke about how being mostly based in children’s centres and/or hybrid working has led to a deterioration in interprofessional communication.

Okay, so the GP calling me, I would fall off my chair, like, genuinely. I wish. In an ideal world the GP and the health visitor would work together, and the GP would say something like, I saw this mum. This baby is really unsettled… (P8, HV)

Often, HVs felt shut out from the diagnosis and treatment process once the GP became involved. Shared electronic medical records were not available to most, leaving some HVs feeling ‘on the back foot’. Most participants also expressed frustration that services were no longer co-located, also contributing to increased barriers to communication between HVs and GPs:

I mean gone are the days where—I mean we’re based in like a primary healthcare centre which is part of a GP’s surgery, but we don’t have any sort of communication with them.

(P7, HV)

From speaking with HV teams across many regions, inconsistency about best practice and resources was clear. There were regional differences in the availability of drop-in clinics, diagnostic flow charts, prevalence of nurses, midwives and HV prescribing and the various roles of the GP and HV. Participants also described inconsistency within their area with practitioners giving different, sometimes conflicting advice.

If they all were speaking off the same hymn sheet, and if we knew for definite that they would go and see the GP and then be referred to a dietitian and would see a dietitian or an allergy specialist at some point early on, that would be really reassuring. Also, just making sure that all the GPs are on the same page, as well, with it; that they all are doing the same thing

(P14, CHN)

### The tipping point model

Stemming from the themes, we developed the tipping point model to conceptualise the narrative which emerged from the participants, in relation to their experiences of supporting families with unsettled babies. This model demonstrates the pathway through which the health visiting service can effectively support families of unsettled babies. However, the negative effects of service and staffing cuts appear to mediate the current state of service delivery, and this leads to a tipping point for staff capacity and well-being, creating a barrier to effective support for families.

## Discussion

This paper highlights how HV teams support families with unsettled babies. Overall, participants reported the importance of their health visiting and nursing roles (in addition to other HCPs) in assessing the baby, signposting to relevant services and providing emotional containment. It was clear that many HVs and CHNs considered that in most cases, babies’ unsettled behaviours were a normal developmental phase and did not require medical intervention. Assessment of this was often reported as a complex process that required time. Containment and reassurance were frequently the strategy required. However, despite the potential of health visiting teams’ input to reduce parental anxiety, prevent unnecessary medicalisation and GP consultations, this was undermined by restrictions in services, changes in service organisation and changes in the way work is allocated within the skill mix teams caused by long-term lack of funding.

Findings regarding lack of staff capacity, cited frequently within the present study (with cutbacks to drop-in services and access to a specialist qualified HV), are in line with recent research. For example, qualitative research in Ireland that focused on improving healthy infant feeding advice^34^ reported that primary health professionals (such as GPs, practice nurses and public health nurses) reported similar barriers, such as overwhelmed services and limited GP resources, and that there is a need for parental support in breastfeeding. In the current study, many HVs believed they could not provide the consistent support a family needs to feel contained, which also mirrors the experiences of the parents summarised in the wider literature.^24 25^

In the present study, the use of technology, extended family and social networks by parents attempting to seek reassurance was often a source of concern to HV teams; many participants reported misinformation given to parents about fourth trimester behaviour, leaving parents feeling like failures, or concerned that there may be something medically wrong with their baby. This reflects the results of a recent systematic review of qualitative research into parent experiences, which proposes that parents’ illness-related interpretations of unsettled baby behaviour may be rooted in feelings of guilt and a desire to construct an identity as a ‘good parent’.^35^

The HV/CHN perception of parental misinterpretation of normal baby behaviours (influenced by societal and cultural influences such as social media), together with difficulties in service (reduced contact with HVs and variation in services), seems to lead parents into uncertainty about the support HVs can offer. Service cuts make navigating a clear pathway difficult, and as a result, parents often bypass HVs to consult with a GP, increasing the risk of medicalisation of baby behaviours. Hornsey *et al*^25^ explored parental experiences of having an unsettled baby and found that parents had mixed experiences of support from health professionals, with many parents feeling dismissed by services. As a result, many parents turn to online support or use of their social networks, again increasing the risk of misinterpretation of baby behaviours. This was also the general viewpoint of parents from an HVs/CHNs perspective in the present study.

### Strengths and limitations

Research with HCP populations in the UK, including health visiting teams, about contemporary management of unsettled baby behaviours and other common baby symptoms is lacking, and this study provides much needed research to illuminate this. A sample size of 20 HVs/CHNs with a variety of nursing experience and length of service within HV teams provided sufficient information power to develop rich themes. However, it is acknowledged that the sample consists mostly of HV professionals who are all white British—a more ethnically diverse sample and the inclusion of more CHNs would add to the information power of this analysis. Providing some examples of unsettled baby behaviours in the recruitment material may have influenced participant responses to the question about what the term ‘unsettled baby’ means to them. Nevertheless, these examples were only briefly provided to facilitate the recruitment stage, whereas the interviews explored the participants’ personal views in more depth. They did not repeat these examples prior to asking this question and participants responded in depth.

### Implications of findings

Findings suggest HV services need to be sufficiently resourced so that parents who need it can access support and advice. This will help prevent parental distress and unnecessary medicalisation of unsettled baby behaviour, potentially reducing health service use and costs. At a minimum, the awareness of what services and support HV teams can offer to parents of unsettled babies must be raised. There is also a need for sufficient staff in the workforce with a specialist HV qualification, and sufficiently low HV to child ratios to allow for continuity, listening visits or containment to be provided. Technology will always be a complement to face-to-face services, and in light of recent reductions in home-visiting and clinic services, it seems likely that it will continue to play an important part in parental management of unsettled babies. However, it is important to ensure that resources are evidence-based. Hybrid/remote working and a move from localised to broader geographical coverage by teams reduces opportunities for more collaborative work across multidisciplinary HCP teams, such as sharing of best practice. Additionally, there was reported inconsistency in services between different Trusts. A universal, evidence-based pathway is recommended, outlining GP and HV roles alongside other, more specialised services and a clear strategy for interprofessional communication. Future research should explore GP experiences of supporting families with unsettled babies to shed further light on the system of HCP support.

## Conclusions

In conclusion, the current study makes an important contribution to the growing literature on unsettled babies and provides a ‘tipping point’ model of how the health visiting service, with appropriate funding, could provide much needed support to families. The model further highlights how funding cuts and service delivery changes have restricted the ability of HVs to provide this support.

### Recommendations

Policy makers and commissioners within the NHS need to agree a universal, evidence-based pathway for management of unsettled babies, including assessment for allergy and reflux, specifying professional roles and interprofessional communication strategies.

Health services also need to fund, commission and organise services to reflect the value and support offered by ‘containment’. This includes the option of home visits and drop-in clinics, delivered to allow for relationship-building between parent and HV.^8^ Training and guidelines could be improved for HVs and CHNs to facilitate the assessment process for unsettled babies.

Finally, there needs to be greater awareness-raising to parents of the pathways and the services that HV teams can offer in the management of unsettled baby behaviour.

## Supplementary material

10.1136/bmjopen-2025-101051 online supplemental file 1

## Data Availability

No data are available to be shared.
