# Supplementary material for: Health visitor and community health nurse perspectives of supporting parents caring for unsettled babies: a qualitative interview study
Source: BMJ Open. 2026 Feb 12;16(2):e101051. doi: 10.1136/bmjopen-2025-101051 (PMC12911685; doi:10.1136/bmjopen-2025-101051)
Supplement: online supplemental file 1 [file bmjopen-16-2-s001.docx]

**Appendix 1 Semi-structured Topic Guide**

| Topic area | Questions | Prompts |
| --- | --- | --- |
| Warm up |  |  |
| Views and experiences of supporting families directly | What does the term ‘unsettled baby’ mean to you? | - What do you think about [ colic (crying)/ vomiting / strange poos / rashes]? - What if anything, did you think caused or make the symptoms worse? - What, if anything, do you feel can help families manage baby symptoms? - If not mentioned, ask specifically about views on excessive crying, milk allergy and reflux - What do you feel the impact is on having an unsettled baby on the parents? - In relation to (the above) how do you feel about this? - What questions do you feel you need to ask of a family to understand their concerns? |
|  | What are your experiences of supporting families with unsettled babies? | - How do you feel when a parent presents with this issue? What is your immediate reaction? Why do you think you feel like this? - What happened? - How do the conversations usually start? - What do these conversations typically centre on? - How do you usually advise parents? - How do you feel they respond to your advice? - What feelings or emotions did you experience? - Did any of these thoughts worry you? - How did you feel when you came away from the contact? |
|  | How do you usually support families with unsettled babies? | - How confident do you feel to support these families? - Is there anything that you feel impacts your ability to support families with unsettled babies? If so, can you tell me about this? - Prompts: anything that helps? Anything that makes it more difficult? - How do you build relationships with the family? - How important is this? - Are there any particular techniques you use when talking to parents about this subject? - Would you tailor the advice or support you offer for individual families?  If so, how? If not, how? - What resources, if any, do you signpost parents to? - What resources, if any, do you use to support your own knowledge or practice in this area? Are they useful? How? How not? - Would support look different outside of the region you serve? - Prompts: how might support look different? - What might be the impact of this? - Have you come across any cultural issues or differences which might affect the support you offer? - Have you found differences in family dynamics that you think are relevant or important? - Are there any wider societal issues that you think are relevant or important? |
|  | What are your views on feeding? | - What are you views on breastfeeding? - What are your views on chest feeding? - What are your views on formula? - Can you tell me about your experiennces in supporting feeding? - How confident do you feel when giving this support? - What role do you think the feeding plays in unsettled behaviours? |
| Parental attitudes | How do you think parents make sense of these symptoms? | - How does this align with your views? - What do you think parents expect from your service? - How well do services meet parents’ expectations? - Why do you think you have/have not been able to meet parental expectations? - How do you think parents feel about coming to you about their baby’s unsettled behaviours? - What do you think the outcome of the contact is? Do you feel able to support families? - Does anything about this concern/worry you? |
| Managing symptoms | If applicable, can you tell me about any training you have received about managing unsettled babies? | - Where did you receive this training? - How helpful was the training? - What support do you receive from the Health Visiting network? - How supported do you feel in your day to day role when supporting unsettled infants? - How well do you think the training you received fits with your perception of how families should be supported? - What are your views on medications or special formula? - What are your views on maternal dietary exclusion? When do you suggest it, if ever? |
|  | Do you give any specific advice do you give parents about their unsettled infants? If so, can you tell me about this? | - Do you think specific advice or soothing strategies are needed? - Do parents want specific advice? - Do you feel comfortable giving specific advice? - Where did you find out about this? - What are your thoughts about this? - Has anything stopped you from giving advice? - How did the advice you were asked to provide make you feel? - Is the advice helpful for parents? Do you feel the parents follow the advice provided? Does that matter to you? - Have you ever prescribed for an unsettled baby? - If so, how did you feel about doing this? |
|  | How able do you feel managing unsettled babies symptoms? | - How has your confidence to support these symptoms changed over time? - What has influenced your confidence level over time? - Why do you feel confident / less confident - Has anything helped you to feel more confident? - Have there been differences in your confidence level with different families? What are they? |
| Accessing information and support from GP’s | When would you refer a family to their GP? | How do parents respond to this?  Why do you think this response happens?  Do you feel confident on when to refer to a GP  How do you feel about referring to a GP |
|  | What are your experiences of GP management of unsettled babies? | - How do you think parents see the role of their GP’s when looking for support for their baby? - How do you think GPs see their role in supporting parents? - Do you know what support families usually receive from GP? - Are you aware of any signposting offered to parents? - What do you think works well in General Practice? - What do think doesn’t work well? - What do you think the role of a GP should be in managing unsettled babies? - Are there any differences in how the GP should be managing these contacts and how a HV should? - How can HVs and GPs work together to support these families best? - Have you had any experience working together with other professionals such as GPs to support these families? If so what worked? What didn’t work? |
| General views about best practice and service changes | What do you think families need to support them with unsettled babies? | - Are there any groups which have more unmet needs than others?   b. What would help to make sure these needs can be met?   - In relation to (b) what could be done to ensure you receive this help? - In relation to (b) do you perceive there to be any barriers to receiving this help?   e. What do you believe the impact of unmet needs might be?   - In relation to (e) does anything worry/concern you? |
|  | What advice would you give to other health visitors supporting families with unsettled babies with these symptoms? | - What advice would you give to other services providers who support families with unsettled babies? - Out of what you have said, can you tell me what advice is most important to you to include? |
|  | Have you experienced any changes to the way the HV service has run? | - How has this impacted the way you support families now? - What has contributed to this change? - Has this change improved support for families? - Within the current service, do you have the time needed to support families? - How does this impact your capacity to support families? - How does this impact the relationships you build with the families? - Is this impactful on the way you provide support? - How does this make you feel? - What (if any) changes would you make to the way the service supports unsettled infants currently? |
|  | Is there any part of managing unsettled babies that you would like more advice about? | - Are there any parts of managing these symptoms that we may not be aware of that are particularly important to you? - Are there any other aspects of your job role that should be taken into consideration? |
