# Supplementary material for: Health visitor and community health nurse perspectives of supporting parents caring for unsettled babies: a qualitative interview study
Source: BMJ Open. 2026 Feb 12;16(2):e101051. doi: 10.1136/bmjopen-2025-101051 (PMC12911685; doi:10.1136/bmjopen-2025-101051)
Supplement: Supplementary file 2 [file bmjopen-2025-101051.reviewer_comments.pdf]

PEER REVIEW HISTORY

BMJ Open publishes all reviews undertaken for accepted manuscripts. Reviewers are asked to complete a checklist review form and are provided with free text boxes to elaborate on their assessment. These free text comments are reproduced below.

ARTICLE DETAILS

Title (Provisional)

Health visitor and community health nurse perspectives of supporting parents caring for unsettled babies: a qualitative interview study

Authors

Smith, Lucy; Hornsey, Samantha Jade; Latter, Sue; Dobson, Amy; Miller, Sascha; Henaghan-Sykes, Kate; Adams, Sue; Santer, Miriam; Muller, Ingrid

---

VERSION 1 - REVIEW

**Reviewer**	**1**
**Name**	**Friedmann, Anna**
**Affiliation**	**Technical University of Munich**
**Date**	**02-May-2025**
**COI**	**None**

I recently had the opportunity to review your manuscript. I believe that it could make a valuable contribution for advising practicioners and health policy makers, however some issues need to be addressed:

Introduction:

l. 21: please give a short defintion of excessive crying when you first use the term.

l. 29 ff.: what is it that causes harm to the families? The misinterpretation or the symptomalogy itself?

Methods:

Inclusion criteria: How was an unsettled baby defined exactly? By whom? This needs to be more precise. I am also not sure that excessive crying and vomiting pose the same risks (e.g., excessive crying being a risk factor for child maltreament...).

Data analysis: Please explain how coding was discussed in the team. Was every code discussed? Why did only one person do the coding as supposed to two persons (interrater reliablilty...)? Which "proportion of transcripts" was read by the team? Please be more precise.

**Reviewer**	**2**
**Name**	**Mardani-Hamooleh, Marjan**
**Affiliation**	**Iran University of Medical Sciences**
**Date**	**13-Aug-2025**
**COI**	**None**

The study is very weak in terms of methodology. The method is not written based on qualitative studies. The design is unclear. The sampling, data analysis, and rigor are very weak.

VERSION 1 - AUTHOR RESPONSE

Reviewer 1

Comment	Response
I recently had the opportunity to review your manuscript. I believe that it could make a valuable contribution for advising practicioners and health policy makers, however some issues need to be addressed:	Thank you for your helpful feedback.
Introduction	
21: please give a short defintion of excessive crying when you first use the term.	Thank you for highlighting the need to include this.<br><br>On page 2 (lines 58-60) of the tracked change version of the main text, we have added the following text:<br><br>"Within this paper, excessive crying is defined as crying that continues for longer than usual for the baby, after repeated attempts have been made by the caregiver to meet their needs".
29 ff.: what is it that causes harm to the families? The misinterpretation or the symptomalogy itself?	This point has been addressed through the addition of the following text on page 2 (lines 61-63) of the tracked change version of the main text:<br><br>"Excessive crying or other infant symptoms are often interpreted as cow's milk allergy (CMA) or gastro-oesophageal reflux; however, there is growing concern that this may cause significant harm to babies and families; overtreatment of symptoms can undermine breastfeeding, lead to added stress and anxiety for the family, and increase risk of negative outcomes and discomfort for the child."
Methods	

Inclusion criteria: How was an unsettled baby defined exactly? By whom? This needs to be more precise. I am also not sure that excessive crying and vomiting pose the same risks (e.g., excessive crying being a risk factor for child maltreament...).	This is an excellent point which highlights the wider lack of clear conceptual definition of this concept within the field. In the recruitment material, we gave some brief examples of behaviours/symptoms unsettled babies may have (e.g. such as "excessive crying" or "vomiting" or "fussing unless being held"). This helped us to recruit eligible health professionals, however we did not give a specific definition of what constitutes an unsettled baby.<br><br>• We added the following on page 3 (lines 103-106) of the tracked change main text: "In the recruitment material, we gave some brief examples of behaviours/symptoms unsettled babies may have (e.g. "excessive crying", "vomiting", "fussing unless being held"). This helped us to recruit eligible health professionals, however due to a lack of clear conceptual definition of this concept, we did not give a specific definition of what constitutes an unsettled baby."<br><br>To gather views about what 'unsettled baby' meant to the participants, we included a question within the interview topic guide which asked participants "what does the term unsettled baby mean to you?", with semi-structured follow up questions as appropriate to elicit rich data from the participants. All at least described that this was a baby who cried excessively.<br><br>• On page 3 (line 128) of the tracked change main text, we made the following edit: "Six main questions asked about (1) beliefs about what the term unsettled baby means to them, including beliefs around unsettled babies' behaviour in relation to symptoms, causes, assessment and treatment (2) experiences of supporting families with unsettled babies..."<br><br>We acknowledge as a limitation that having this information in the recruitment material may have influenced the participant's response in the interview. However, this was to facilitate recruitment and was not repeated prior to asking this question in the interview. Participants also responded to the interview questions in great detail, more so than our brief examples of behaviours/symptoms prior.<br><br>We have therefore add this to the limitations on page 12 (lines 433-437) of the tracked change version of the main text.

	• "Providing some examples of unsettled baby behaviours in the recruitment material may have influenced participant responses to the question about what the term 'unsettled baby' means to them. Nevertheless, these examples were only briefly provided to facilitate the recruitment stage, whereas the interviews explored the participants' personal views in more depth. They did not repeat these examples prior to asking this question and participants responded in depth"
Data analysis: Please explain how coding was discussed in the team. Was every code discussed? Why did only one person do the coding as supposed to two persons (interrater reliablilty...)? Which "proportion of transcripts" was read by the team? Please be more precise.	Thank you for highlighting the need for further clarification. One person conducted all of the initial coding framework. The team read all interview summaries and 3 members of the team each read 2 transcripts in order to aid interpretation of the coding, interpretation and analysis. We did not aim for interrater reliability because Reflexive Thematic Analysis (Braun and Clarke 2019), rather than aiming for structured reliability/agreement between coders, places more purpose on researcher reflexivity, engagement with their data and a collaborative approach to development of themes. We have made the following changes for greater transparency.<br><br>Page 4 (lines 139-148): "All interview transcripts were analysed inductively using Reflexive Thematic Analysis [32]. This allowed for a flexible and pragmatic approach to data analysis with the paradigmatic framework of constructivism and interpretivism. Data were handled using NVivo v14 software. LS carried out the initial coding framework and all coding was discussed with all members of the team, who fed back. LS iterated the coding based on feedback, and this was again circulated to the whole team for input. The team read the interview summaries, and IM (female, professor and qualitative expert), AD (female, PhD student and health visitor, qualitative researcher) and SH (female, post-doctoral researcher, experienced qualitative researcher) each further read two separate transcripts to aid development of coding, interpretation and analysis. The team were happy with the codes/themes, developed ~~Each transcript was coded by LS and discussed with the team (IM, SH, MS, AD, SL, SA, KHS and SM). in the research team. Following discussion, codes were further refined. Themes were developed~~ through team discussion and in partnership with public contributors~~The team read a proportion of transcripts and interview summaries to aid development of coding, interpretation and analysis.~~ It was not within the

	capacity of this project to return transcripts/themes to participants…”

Reviewer 2

Comment	Response
The study is very weak in terms of methodology. The method is not written based on qualitative studies. The design is unclear. The sampling, data analysis, and rigor are very weak.	Thank you for your feedback, we have realigned the methods in line with the COREQ guidelines, instead of SRQR which we hope provides more clarity, as indicated in the track changes.<br><br>We have added additional text to fulfil the COREQ guidelines (as listed below) and additional text throughout the manuscript in response to feedback from the editor and reviewer 1 (for example further details on analysis). These have all been marked as tracked changes.<br><br>Additions of text based on the COREQ:<br>• Page 3, line 94-95 – replaced SRQR reporting checklist citation with COREQ reporting checklist citation.<br>• Page 3, lines 107-109. Added “We are not aware of who decided not to express an interest because we did not know who the study was advertised to unless they expressed an interest to us after seeing the recruitment materials.”<br>• Page 4 line 122: “LS (a female post-doctoral researcher with experience in qualitative research methods) conducted the interviews and did not know the participants prior to the interview and presented herself as neutral and non-clinical to the participants”<br>• Page 4 line 124-125: Added “There were no nonparticipants present at the interview”<br>• Page 4 line 149. Add “It was not within the capacity of this project to return transcripts/themes to participants for checking”
